# Multiple anthropogenic interventions drive puma survival following wolf recovery in the Greater Yellowstone Ecosystem

**DOI:** 10.1002/ece3.4264

**Published:** 2018-06-25

**Authors:** L. Mark Elbroch, Lucile Marescot, Howard Quigley, Derek Craighead, Heiko U. Wittmer

**Affiliations:** ^1^ Panthera New York New York; ^2^ School of Biological Sciences Victoria University of Wellington Wellington New Zealand; ^3^ Craighead Beringia South Kelly Wyoming

**Keywords:** apex predators, biodiversity, competition, hunting, population dynamics, reintroductions

## Abstract

Humans are primary drivers of declining abundances and extirpation of large carnivores worldwide. Management interventions to restore biodiversity patterns, however, include carnivore reintroductions, despite the many unresolved ecological consequences associated with such efforts. Using multistate capture–mark–recapture models, we explored age‐specific survival and cause‐specific mortality rates for 134 pumas (*Puma concolor*) monitored in the Greater Yellowstone Ecosystem during gray wolf (*Canis lupus*) recovery. We identified two top models explaining differences in puma survivorship, and our results suggested three management interventions (unsustainable puma hunting, reduction in a primary prey, and reintroduction of a dominant competitor) have unintentionally impacted puma survival. Specifically, puma survival across age classes was lower in the 6‐month hunting season than the 6‐month nonhunting season; human‐caused mortality rates for juveniles and adults, and predation rates on puma kittens, were higher in the hunting season. Predation on puma kittens, and starvation rates for all pumas, also increased as managers reduced elk (*Cervus elaphus*) abundance in the system, highlighting direct and indirect effects of competition between recovering wolves and pumas over prey. Our results emphasize the importance of understanding the synergistic effects of existing management strategies and the recovery of large, dominant carnivores to effectively conserve subordinate, hunted carnivores in human‐dominated landscapes.

## 1 INTRODUCTION

Humans are primary drivers of declining abundances and subsequent extirpation of large carnivores worldwide (Faurby & Svenning, 2015; Ripple et al., 2014). Causes for observed declines are varied but include habitat loss and fragmentation, declining prey populations, and, in particular, direct killing by humans (Ripple et al., 2014; Treves & Bruskotter, 2014; Wolf & Ripple, 2016). Humans primarily kill large carnivores because of real and perceived threats to property and human safety, competition for shared resources, and following complex social norms supporting poaching and trophy hunting (Darimont, Codding & Hawkes, 2017; Elbroch, Feltner & Quigley, 2017; Treves & Bruskotter, 2014). Regardless of human motivations, large carnivores are particularly susceptible to human‐caused mortalities due to slow life histories that include a reliance on adult female longevity and long interbirth intervals (Darimont, Fox, Bryan & Reimchen, 2015). Without management interventions that aid carnivores, many apex predators are predicted to decline to extinction (Ripple et al., 2014; Wolf & Ripple, 2016).

Management strategies to conserve and restore large carnivores include reintroduction and translocation efforts (Seddon, Griffiths, Soorae & Armstrong, 2014). These strategies are mostly implemented in Europe and North America, where increasing tolerance may allow viable populations of large predators to exist in human‐dominated landscapes (Chapron et al., 2014). Studies following the recovery of large carnivores have also made important contributions to our current understanding of the direct and indirect effects of predators upon ecological communities, including predator–prey interactions and trophic cascades affecting community assemblages (e.g., Ripple & Beschta, 2011). The effect of recovering carnivores on other, subordinate, carnivores has received much less attention. This is surprising given known effects of competition within carnivore guilds, including reduced abundance and survivorship among subordinate carnivores (Harihar, Pandav & Goyal, 2011; Levi & Wilmers, 2012; Roemer, Donlan & Courchamp, 2002) and shifts in space use or prey selection by subordinate carnivores (Harihar et al., 2011; Lendrum et al., 2014; Ruth et al., 2011).

The reintroduction of wolves (*Canis lupus*) to Yellowstone National Park, USA, began in 1995 and is touted as one of the most successful conservation stories of all time (Smith & Ferguson, 2012). Most research following the reintroduction of wolves focused on their effect on prey populations, particularly elk (*Cervus elaphus*; e.g., Vucetich, Smith & Stahler, 2005; Eberhardt, White, Garrott & Houston, 2007). Wolves, however, are also expected to impact resident carnivores.

Pumas (*Puma concolor*) are solitary carnivores and, like wolves, important components of ecological communities (Allen, Elbroch, Wilmers & Wittmer, 2015; Elbroch, Peziol, O'Malley & Quigley, 2017). They are also a conservation success story, in that puma populations rebounded in the west of the United States and Canada after 1965, when wildlife managers in nearly every western state stopped paying state bounties for killing pumas and introduced managed puma hunting with limits in restricted seasons (Mattson & Clark, 2010). Previous research has suggested that pumas are subordinate competitors in the presence of wolves (Kortello, Hurd & Murray, 2007; Ruth, 2004), but research has failed to demonstrate clear fitness consequences for pumas competing with wolves despite numerous recorded observations of wolves killing pumas (e.g., Kunkel, Ruth, Pletscher & Hornocker, 1999; Ruth et al., 2011). Pumas are also a game species legally hunted throughout much of their range. High hunting pressure can severely reduce the abundance of pumas and destabilize established population structures (Robinson, Wielgus, Cooley & Cooley, 2008; Stoner et al., 2013). Thus, while direct fitness consequences of recovering wolf populations on pumas are likely, demonstrating them requires data and analytical approaches capable of separating their potential effects from those attributable to other limiting factors, such as hunting.

Our objective was to test for the effects of recovering wolves on puma survival in the southern Greater Yellowstone Ecosystem (GYE), where existing management objectives also supported the legal hunting of pumas and decreasing the local elk population through “liberal” hunting seasons (WGFD, 2014). To achieve our objective, we used 14 years of monitoring data from 134 individually marked pumas collected concurrent with the recolonization of wolves and elk reduction to determine drivers of puma survival and cause‐specific mortality rates.

## 2 MATERIALS AND METHODS

### 2.1 Study area and wolf reintroductions

Our study area encompassed approximately 2,300 km^2^ of the GYE in southern Teton County, Wyoming (Appendix S1, Figure A1.1). The area was constrained by Yellowstone National Park to the north, Grand Teton National Park to the west, and the National Elk Refuge to the south and supported an abundance of ungulate prey, both in terms of species (*n *=* *7) and biomass. Detailed descriptions of climate, topography, habitat, and community composition including ungulate prey species are presented in Elbroch, Lendrum, Newby, Quigley and Craighead (2013).

Wolves were first reintroduced north of our study area in Yellowstone National Park in 1995 (Smith & Ferguson, 2012). The first breeding pair settled in our study area in 1999, where annual estimates for the numbers of wolves and wolf packs in the study area were determined by the United States Fish and Wildlife Service (USFW; Appendix S2, Table A2.1). Wolves were protected from legal hunting during our study excepting 2012 and 2013, when a limited quota hunt was permitted from October 1 to December 31 of each year.

Elk in our study area were part of the migratory Jackson herd and cooperatively managed by the Wyoming Game and Fish Department (WGFD), National Park Service, and the USFW's National Elk Refuge (NER). The Jackson elk herd typically travels long distances and congregates adjacent the town of Jackson, WY, where they receive supplemental feeding in winter on the NER and adjacent public lands on feed lots managed by the WGFD. Our study covered the time period in which managers implemented liberal hunting quotas across jurisdictional boundaries to reduce the Jackson herd from 16,000 in 2000 to 11,000 animals, of which managers wanted 5,000 to winter on the NER (Cole et al., 2015; U.S. Fish and Wildlife Service & National Park Service, 2007; WGFD, 2016).

### 2.2 Puma captures, monitoring, and age classifications

Puma monitoring began in 2001 and we followed puma capture and immobilization protocols described in Elbroch et al. (2013) and approved by the Jackson Institutional Animal Care and Use Committee (Protocol 027‐10EGDBS‐060210). We fit pumas with a VHF (Telonics, Mesa, AZ) or GPS (Telonics, Mesa, AZ; Televilt, Lindesberg, Sweden; Vectronics, Berlin, Germany; Lotek Wireless, Ontario, Canada) collar. We hand‐captured kittens between 5 and 7 weeks old without the aid of immobilization drugs and fit them with custom‐made, lightweight, expandable VHF collars (Telonics, Mesa, AZ, USA).

Attempts to locate kittens wearing VHF collars were made every 2 days. All other pumas wearing VHF collars were located at minimum weekly from the ground and monthly from aircraft. Location data were acquired by GPS collars 4–12 times per day. All collars were equipped with mortality sensors, which alerted researchers when an individual had not moved for ≥8 hr. We investigated mortality sites and determined the cause of death through interpreting field signs (e.g., bite marks, footprints), necropsies conducted with a veterinarian, and based on blood and tissue samples analyzed by the Wyoming State Veterinary Laboratory.

### 2.3 Determining minimum annual puma densities

Each year, we determined minimum puma density in our study area based on overlapping home ranges (Rinehart, Elbroch & Wittmer, 2014). Annual home ranges for adult pumas were determined using fixed‐kernel density estimators (Worton, 1989) in ArcGIS 10, and isopleth calculations in the Geospatial Modeling Environment (Beyer, 2012); methods are further described in Lendrum et al. (2014).

We determined the boundaries of the area in which we consistently searched for pumas each winter, and in which we believed we had captured all resident pumas. In ArcGIS 10, we created a polygon of our capture area and quantified each puma's residency within this polygon (Rinehart et al., 2014). “Minimum puma densities” for our 890 km^2^ capture area were then determined by summing the residency estimates for all adult pumas with overlapping home ranges for each year. We also scaled density estimates to pumas per 100 km^2^ for comparisons with previous research, aware that scaling introduces extrapolation bias impacting the precision of estimates (Schonewald‐Cox, Azari & Blume, 1991; Smallwood & Schonewald, 1998).

### 2.4 Puma survival analyses

We estimated puma survival probabilities and cause‐specific mortality rates (*m*_*i*_; Schaub & Pradel, 2004; Marescot, Forrester, Casady & Wittmer, 2015) with multistate capture–mark–recapture (CMR) models in E‐SURGE (Choquet, Rouan & Pradel, 2009; Lebreton, Nichols, Barker, Pradel & Spendelow, 2009). CMR models were best suited for analyzing our data because of their ability to account for both right and left censored data and to accommodate encounter histories based on different data sources (e.g., kittens observed in dens, individuals monitored with VHF vs. GPS technology) and sampling intervals (Cubaynes et al., 2014; Devineau et al., 2010). However, standard CMR models have to meet multiple assumptions to avoid biasing parameter estimates and model overparameterization (Choquet, Lebreton, Gimenez, Reboulet & Pradel, 2009; Fletcher et al., 2012). Therefore, prior to final model selection, we employed a range of goodness‐of‐fit (GOF) tests to determine if our data met the assumptions for CMR models, to test whether our methods may have biased estimates of survival or recapture probabilities, and to determine whether our models fit the data and explained variation in our selection parameter, puma survival (Appendix S3). We also tested for potential overdispersion in our data due to siblings in the same litter dying from the same cause more than expected under the assumptions of independence (Ruth et al., 2011), and subsequently adjusted AIC values using the variance inflation factor (ĉ; Appendix S3).

For our analyses, we categorized pumas into three age classes based on differences in life histories and survival reported in the literature. We defined kittens (*n* = 75) as individuals <6 months old. Kittens are completely dependent on their mothers and experience high mortality from both predation and starvation (Logan & Sweanor, 2001; Ruth et al., 2011). We defined juveniles (*n* = 22) as individuals ≥6 and <18 months old. Juveniles remain dependent on their mothers, but are less susceptible to predation (they better avoid predators by climbing or running); (Logan & Sweanor, 2001)). Juveniles experience higher risks of starvation, can be legally hunted once they are one year old and separate from their mothers (WGFD, 2006), and experience risks associated with dispersal (Quigley & Hornocker, 2010; Stoner et al., 2013). We pooled all individuals ≥18 months old into an adult age class (*n* = 37) when pumas are expected to establish stable territories and become reproductively active (Logan & Sweanor, 2001; Quigley & Hornocker, 2010).

At each time step of the model, individuals (i.e., kittens, juveniles, adults) occupied one of the following seven states: “*alive*” (survival rate ϕ in matrix below); “*recently dead from hunting, poaching, or management action*” (i.e., human causes; cause‐specific mortality rate *m*_*h*_); “*recently dead from predation by wolves, bears (Ursus spp.), or other pumas*” (cause‐specific mortality rate *m*_*p*_; the small number of predation events limited our ability to further differentiate between predator species); “*recently dead from starvation*” (cause‐specific mortality rate *m*_*s*_); “*recently dead from other natural causes including disease and exposure during cold weather*” (cause‐specific mortality rate *m*_*o*_); or “*recently dead from unknown causes*” (cause‐specific mortality rate *m*_*u*_). All dead individuals were eventually assigned to a permanent “*dead*” state independent of their actual cause of mortality (Lebreton, Almeras & Pradel, 1999). We also censored emigrating pumas (i.e., “*alive outside the study area*”) from these analyses, meaning that they were only included until their departure from the study site, ignoring their subsequent fates. Censoring dispersers in this way mitigated an inflation in mortality estimates due to a reduced sample size, as well as allowed us to quantify mortality rates specific to our study population. The survival transition matrix S from time *t* to time *t *+* *1 was written as

$$S\;=\;\left[\begin{array}{ccccccc} \phi & m_{h} & m_{p} & m_{s} & m_{o} & m_{u} & 0\\ 0 & 0 & 0 & 0 & 0 & 0 & 1\\ 0 & 0 & 0 & 0 & 0 & 0 & 1\\ 0 & 0 & 0 & 0 & 0 & 0 & 1\\ 0 & 0 & 0 & 0 & 0 & 0 & 1\\ 0 & 0 & 0 & 0 & 0 & 0 & 1\\ 0 & 0 & 0 & 0 & 0 & 0 & 1 \end{array}\right].$$

The matrix could further be decomposed into two equivalent biological processes, survival and its associated probability ϕ, and the probability of mortality by a specific cause $m_{i}$, as ϕ is equal to the complementary of the sum of mortality rates (Schaub & Pradel, 2004).

We began model building by identifying biologically relevant covariates that might explain change in puma survival and cause‐specific mortality rates, from which we built competing a priori models to test in an information theoretic framework (Burnham & Anderson, 2002). We also tested whether recapture/detection probabilities were time‐dependent (Culina, Lachish, Pradel, Choquet & Sheldon, 2013), before building candidate models from puma covariates, including age (i.e., kittens, juveniles, adults), and additional ecological and time‐based covariates described below.

We included the following numeric covariates in our models: (a) annual wolf counts for our study area as reported by the USFW, (b) annual counts for the Jackson elk herd as reported by WGFD, (c) annual counts of elk in the Jackson elk herd that wintered off the National Elk Refuge (NER) as reported by the WGFD and NER, (d) annual puma harvest numbers for Unit 2, the hunting unit in which we studied pumas, and (e) annual minimum puma densities, calculated as described above (resident adults/890 km^2^; Appendix S4, Table A4.1). We used two elk metrics as a measure of prey availability and bottom‐up effects, as elk were the primary prey for pumas in our study area (Elbroch et al., 2013): first, we employed total elk numbers in the Jackson herd, and second, the portion of the Jackson elk herd off‐Refuge, to highlight those elk more likely truly available to pumas in our study (Elbroch, Lendrum, Robinson & Quigley, 2016). We excluded density/abundance estimates of wolves, pumas, and elk for 2001 and 2015 from the analysis because puma capture efforts did not occur throughout these years.

We also included two time‐based covariates that captured greater ecological complexity than numeric covariates without overparameterizing models; time‐based covariates, however, also introduced complexity when interpreting results. First, we tested for potential seasonal variation in survival probabilities (*ϕ*) and cause‐specific mortality rates to account for environmental variability due to weather, prey availability, puma foraging behaviors (Elbroch et al., 2013), and anthropogenic top‐down effects, primarily human hunting (Wyoming Game and Fish Department, 2006). We split each year into two 6‐month seasons, following legal hunting periods for pumas, which we expected to yield differences in age‐specific survival and mortality rates because human‐caused mortality is generally the driver of puma population dynamics across hunted populations (Quigley & Hornocker, 2010; Stoner et al., 2013): (a) we defined the “hunting season” as 1 October to 31 March of the following year, during which pumas were legally hunted. The hunting season also captured the following additional ecological variation: elk returned to low‐elevation winter ranges in November and aggregated in large herds near supplementary feeding stations, deer migrated out of the study area, competition between wolves and pumas likely increased near shared prey, and deep snows and cold temperatures increased the risk of starvation (Elbroch, Lendrum, Newby, Quigley & Thompson, 2015; Elbroch et al., 2013); (b) We defined the “nonhunting season” as 1 April–30 September, during which puma hunting was closed, and during which elk migrated to summer ranges at higher elevations and became more widely dispersed, deer returned becoming an integral part of local puma diets, temperatures warmed, and ungulate and puma parturitions occurred (Elbroch, Lendrum, Alexander & Quigley, 2015; Elbroch, Lendrum, Newby et al., 2015).

For our second time‐based covariate, we divided our study into two time periods, primarily reflecting “low‐wolf” and “high‐wolf” densities. We selected a cut‐off date of December 31, 2005, as from 2005 to 2006, the local wolf population doubled in number, initiating a steep period of wolf population growth (Appendix S2, Table A2.1). Elk numbers were highest in the “low‐wolf” period, and more variable, but generally declining during the “high‐wolf” period. The low‐wolf period was also characterized by high human harvest of pumas, and the high‐wolf period by substantially lower but variable human harvest of pumas.

To mitigate issues to do with overparametizing models and including uninformative parameters (Arnold, 2010; Burnham & Anderson, 2002), and to more directly test our specific hypotheses, we devised a short list of competing a priori models (Table 1). All models included one parameter for detection probability, five parameters representing the interaction effects of different cause‐specific mortalities, and five parameters for initial state probabilities (Marescot et al., 2015; Schaub & Pradel, 2004); each model also included the parameters associated with additional numeric (e.g., annual elk counts) or categorical (e.g., seasons, age) covariates. Then we tested which model best fit our selection parameter, puma survival, through a comparison of QAIC_c_ corrected for slightly overdispersed data. We considered the top model and any subsequent model differing by <4 QAIC_c_ units to have produced substantial empirical support for explaining variation in puma survival and cause‐specific mortality rates (Burnham & Anderson, 2002).

**Table 1:** Model selection results for our a priori CMR models estimating survival (*ϕ*) and cause‐specific mortality rates (*m*) for human‐caused, predation, starvation, other, and unknown mortalities of pumas in the Southern Yellowstone Ecosystem. Recapture probabilities (*p*) were constant (*i*) given that models accounting for temporal variation in detection were nonidentifiable. Covariates included annual wolf counts (*Nwolf*), annual counts of the Jackson elk herd (*Nelk*), annual counts of the Jackson herd that wintered off the National Elk Refuge (*NelkOff*), annual puma abundances (*Npuma*), annual pumas harvested in Hunting Unit 2 (*Nharvest*), and two time‐dependent covariates: 2001–2005 versus 2006–2015, reflecting changes in wolf density (*low/high*), and a seasonal comparison based upon the 6‐month hunting season for pumas (*hunt/no‐hunt*). All models estimated age‐specific differences (kitten, juvenile, adult) in survival probabilities and cause‐specific mortality rates of resident pumas across years, and here we report model descriptions: the number of parameters (*n*), deviance, QAIC_c_, ∆QAIC_c_, and QAIC_w_

Cause‐specific mortality models	*n*	Deviance	QAIC_c_	ΔQAIC_c_	Likelihood	QAIC_w_
*ϕ*_age_(hunt/no‐hunt), *m*_age_(hunt/no‐hunt), *p*(*i*)	26	1,344.17	1,398	0	1	0.58
*ϕ*_age_(NelkOff), *m*_age_(NelkOff), *p*(*i*)	26	1,345.31	1,400	2	0.37	0.21
*ϕ*_age_(Npuma), *m*_age_(Npuma), *p*(*i*)	26	1,348.67	1,402.50	4.5	0.11	0.06
*ϕ*_age_(Nelk), *m*_age_(Nelk), *p*(*i*)	26	1,344.36	1,402.50	4.5	0.11	0.06
*ϕ*_age_(low/high), *m*_age_(low/high), *p*(*i*)	26	1,349.47	1,403.30	5.3	0.07	0.04
*ϕ*_age_(Nharvest), *m*_age_(Nharvest), *p*(*i*)	26	1,350.79	1,404.50	6.5	0.04	0.02
*ϕ*_age_(Nwolf), *m*_age_(Nwolf), *p*(*i*)	26	1,379.94	1,433.82	35.82	0.00	0.00
*ϕ*(*i*), *m*(*i*), *p*(*i*)^a^	11	1,499.94	1,553.47	155.47	0.00	0.00

*Note*

a Null model.

## 3 RESULTS

### 3.1 Puma densities

We monitored 134 individual pumas (75 kittens, 22 juveniles, 37 adults) and estimated minimum annual puma densities based on 4.6 ± 1.8 *SD* adult pumas monitored each year. Adult puma densities varied between 2.6 and 8.9 resident adults in our 890 km^2^ capture area, or 0.29–1.0 adults/100 km^2^ (Appendix S4, Table A4.1).

### 3.2 Puma survival analyses

We identified the cause of mortality for 80 of 108 pumas that died during our study; seven died outside the study area and their fates were censored from the cause‐specific mortality analysis. Predominant causes of mortality varied among age classes (Table 2). Predation was the predominant cause of mortality for kittens (9 were killed by wolves), starvation was the predominant cause of mortality for juveniles, and adults most frequently died from human causes.

**Table 2:** Distribution of cause‐specific mortalities among age classes, and across the hunting and nonhunting seasons. “Other causes” included natural mortality, primarily disease and exposure. Causes of mortality of pumas that died outside the study area are shown in parentheses “()” but were censored from analyses

	Human = 18 (7)	Predation = 24	Starvation = 21	Other = 10	Unknown = 28
Kittens	3	13	11	6	16
Subadults	3+ (1)	4	4	2	8
Adults	12+ (6)	7	6	2	4
Hunt	13+ (6)	20	15	9	19
No‐hunt	5+ (1)	4	6	1	9

Our CMR analysis inclusive of cause‐specific mortalities resulted in two top models (Table 1). Our top‐ranked model highlighted differences in age‐specific (kittens, juveniles, adults) variation in survival and cause‐specific mortality rates between hunting and nonhunting seasons (Table 3). This model received 2.8 times more empirical support than our second model based on an evidence ratio (Burnham, Anderson & Huyvaert, 2011). We also assessed age‐specific survivorship among pumas, defined as the complementary of the sum of mortality rates estimated in our analyses. This method assumes mortality rates are additive, which we could not confirm for certain, however, previous research has shown that pumas do not reliably exhibit compensatory mortality under hunting pressure, as would be expected for a territorial species (Cooley et al., 2009). Kitten survival was poor in both seasons, but much lower in the hunting season (0.28 vs. 0.44 in the nonhunting season). Juvenile survival was similar across seasons (0.85 vs. 0.87 in the nonhunting season), although human‐caused mortalities were higher in the hunting season. Adult survival was lower in the hunting season (0.82 vs. 0.89 in the nonhunting season), predominantly due to differences in human‐caused mortality rates. Starvation was higher in all age classes during the hunting season (Table 3).

**Table 3:** Cause‐specific mortality rates attributable to human, starvation, predation, other, and unknown causes. Estimates and standard error in brackets were adjusted using the Heisey and Fuller (1985) method for three age classes of pumas resident in the study area, during the hunting and nonhunting seasons

Season	Cause of mortality	Kitten	Juvenile	Adult
Hunting	Human	0.02 (0.01)	0.05 (0.04)	0.07 (0.01)
	Starvation	0.11 (0.05)	0.07 (0.03)	0.04 (0.01)
	Other	0.02 (0.02)	0.01 (0.01)	0.00 (0.00)
	Predation	0.31 (0.08)	0.02 (0.02)	0.04 (0.01)
	Unknown	0.26 (0.07)	0.00 (0.00)	0.03 (0.00)
No‐hunting	Human	0.02 (0.00)	0.01 (0.01)	0.02 (0.01)
	Starvation	0.01 (0.04)	0.05 (0.03)	0.03 (0.01)
	Other	0.12 (0.05)	0.06 (0.04)	0.01 (0.01)
	Predation	0.16 (0.05)	0.01 (0.01)	0.02 (0.01)
	Unknown	0.25 (0.06)	0.00 (0.00)	0.03 (0.00)

**Figure 1:**
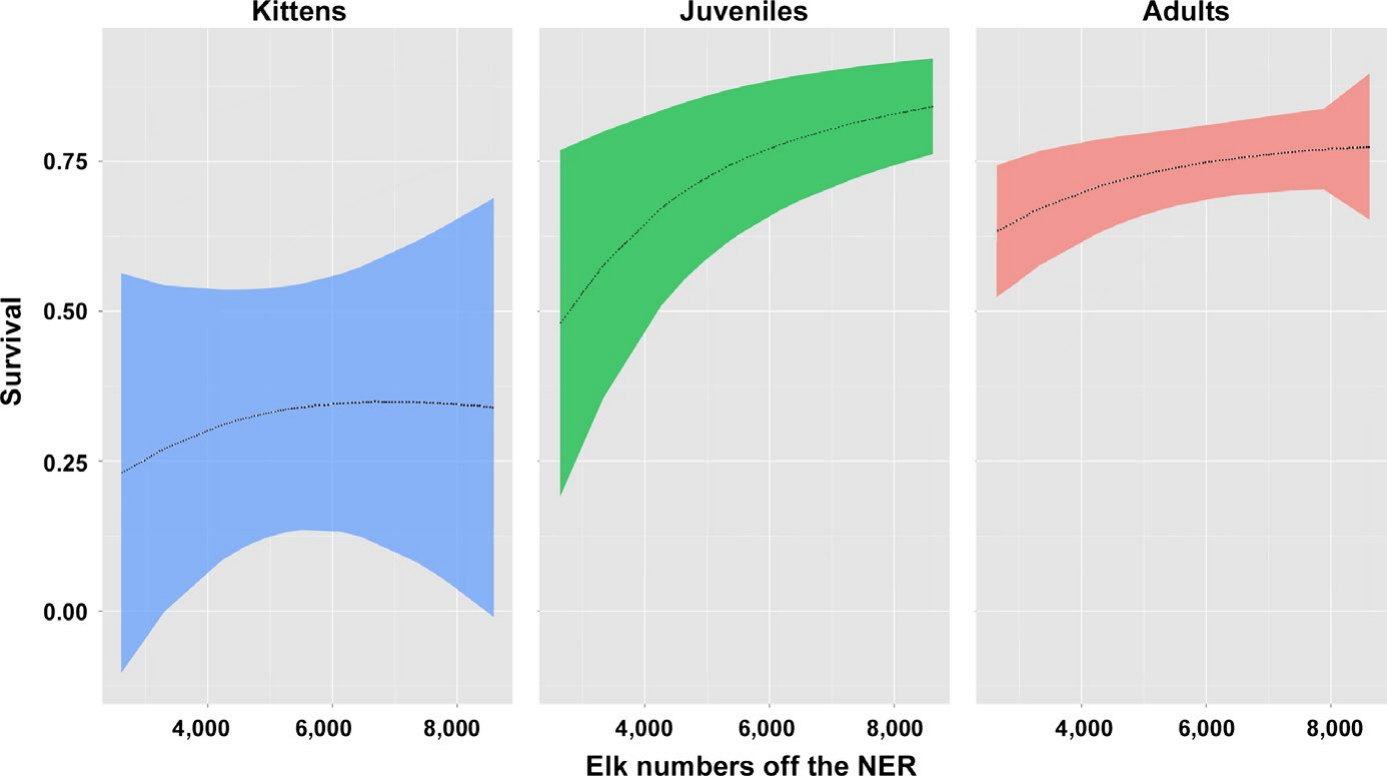
The relationship between elk numbers off the National Elk Refuge (NER) and age‐specific puma survival

**Figure 2:**
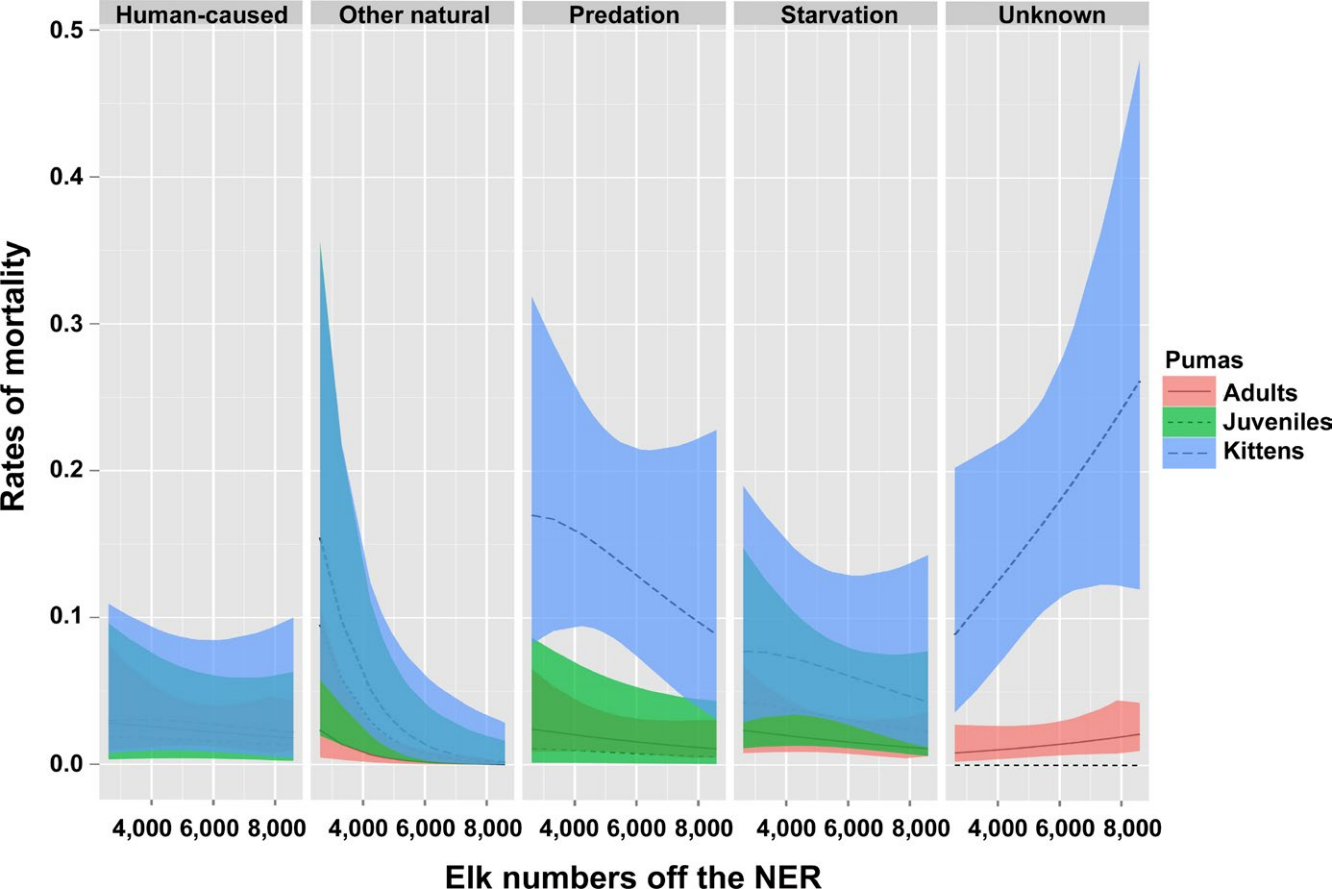
Changes in age‐ and cause‐specific mortality rates with variable elk numbers off the National Elk Refuge (NER)

Our second top model emphasized additional insights missed in the first model, and age‐specific variation in survival and cause‐specific mortality rates correlated with changes in the number of Jackson elk wintering off the NER. All age classes of puma decreased in survival with decreasing elk availability off the NER, but juveniles were impacted most (Figure 1). As elk wintering off the NER decreased, starvation across age classes, other natural causes of death such as exposure and disease across age classes, and predation on kittens increased (Figure 2).

## 4 DISCUSSION

The reintroduction and recovery of large carnivores inside and outside of protected areas are as much a social as an ecological triumph (Chapron et al., 2014). Our research, however, highlighted unanticipated direct and indirect effects of wildlife management manipulating top‐down and bottom‐up forces on a subordinate, resident apex predator, further magnified by the recovery of a top predator. Specifically, our results provided evidence that three management interventions (unsustainable puma hunting, reduction in a primary prey, reintroduction of a dominant competitor) unintentionally impacted subordinate puma survival outside protected areas in the southern GYE. These results highlight the importance of understanding the synergistic effects of existing management strategies and the recovery of large, dominant carnivores to effectively conserve subordinate, hunted carnivores in human‐dominated landscapes.

The first management intervention influencing puma survival was the direct killing of pumas, moderated primarily through managed puma hunting. Our top CMR model identified seasonal differences in puma survival, and we believe our seasonal cause‐specific mortality rates provided insights necessary to disentangle the multiple ecological variables that differed across seasons. Specifically, human‐caused mortality rates in the hunting season were 3.5 times higher for adult pumas and 4.0 times higher for juveniles, as compared with the nonhunting season. Hunting is the primary cause of mortality for pumas throughout the western United States and Canada (Quigley & Hornocker, 2010), and thus these results were not unexpected. Higher kitten and juvenile starvation in the hunting season may also have been influenced by the harvest of females that orphaned kittens too young to forage and defend themselves (Wyoming Game and Fish Department, 2006). Hunting limits for pumas were reduced over the course of our study, however, further reductions in Unit 2 or additional reductions in adjacent hunting units to increase immigration rates (sensu Robinson et al., 2008) may be necessary to facilitate stability in the local puma population.

The second anthropogenic influence on puma survival was the reduced availability of elk, their primary prey (Elbroch et al., 2013). Top predators are generally regulated by prey availability (Wallach, Izhaki, Toms, Ripple & Shanas, 2015), and in our system it was juvenile survival that was impacted most, followed by adult puma survival (Figure 1). As elk decreased, starvation across age classes and predation on kittens both increased (Figure 2). Decreasing elk availability over the course of our study was likely influenced by three contributing factors: first, the Jackson herd was actively reduced following management objectives; second, over the course of our study, a larger proportion of the remaining Jackson herd wintered on the NER (Cole, 2017; WGFD 2016), where they were less available to pumas because of the NER's open expanses without cover and the presence of wolves and people (Elbroch et al., 2016); third, re‐established wolves likely limited elk availability and accessibility through exploitive and interference competition (Elbroch, Lendrum, Newby et al., 2015; Kortello et al., 2007), discussed further below. State wildlife managers, in fact, speculated that elk shifted their distributions to the NER, where they were less available to pumas, due to a combination of wolf predation and earlier winter snowfalls (WGFD, 2016).

The third management intervention influencing puma survival was the reintroduction of wolves to Yellowstone National Park; the recolonization of their descendants resulted in direct and indirect effects on puma survival in our study area. Direct wolf predation on kittens was evident in a comparison of seasonal cause‐specific mortality rates. Kittens experienced extremely high predation rates in the hunting season (0.31 vs. 0.16 in the nonhunting season; Table 3). Every instance of predation by wolves on puma kittens, but one, occurred during winter, and predation by wolves was four times higher than that of infanticide by male pumas during the hunting season. Indirectly, wolves also likely limited puma access to elk through competition (e.g., through reducing elk numbers directly and indirectly; Christianson & Creel, 2014; through kleptoparasitism of puma kills and harassment of pumas at kills; Bartnick, Van Deelen, Quigley & Craighead, 2013; Elbroch, López‐González, Fitzgerald, Kusler & Quigley, 2017c; and through influencing the distribution of elk aggregations on and off the NER, as they move to exhibit dilution in an area with increased predation risk; Brennan, Cross & Creel, 2015; WGFD, 2016). Through these varied competition mechanisms, wolves likely contributed to increased puma starvation across age classes, as has been observed following wolf recolonization in other areas (Kortello et al., 2007; Ruth, 2004).

**Figure 3:**
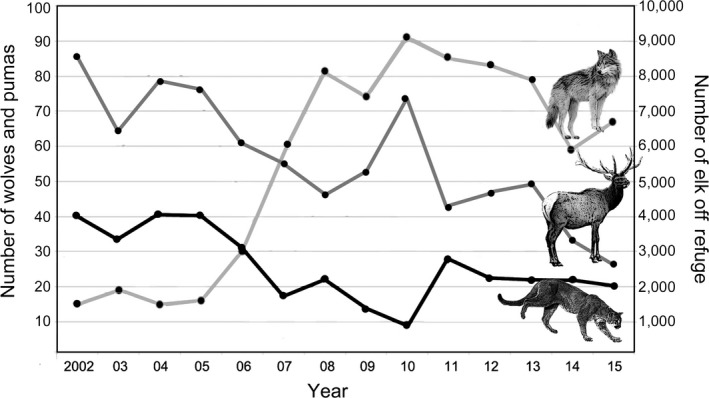
Annual wolf counts, elk numbers off the National Elk Refuge (NER), and estimated number of pumas for our study across our approximately 2,300 km^2^ study area (see Appendix S4)

The magnitude of the emergent effects of top‐down and bottom‐up effects on puma survivorship that we observed was unanticipated, highlighting risks of rapid declines in subordinate, hunted carnivore densities—and their associated ecological functions—following reintroductions of dominant carnivores in managed systems. Adult puma densities dropped 48% from 2002 to 2015 and are now much lower than typically reported for the western United States (mean 0.42 ± 0.07 resident adult pumas/100 km^2^ in our study area from 2011 to 2015 vs. 1.7/100 km^2^ typically reported; Beausoleil, Koehler, Maletzke, Kertson & Wielgus, 2013). Over the same time period, elk availability off the NER dropped by 70% and wolf numbers increased >600% (Figure 3). These results suggest that humans—through their influence on top‐down and bottom‐up forces—have successfully facilitated a change from a system dominated by pumas since 1926, when wolves became extirpated (Haines, 1996) to one dominated by wolves, as in historic times. Given the differences between pumas and wolves in social organizations (solitary felid vs. social canid), hunting behaviors (ambush vs. cursorial), and species‐specific carrying capacities (lower vs. higher), changing relative predator densities is likely to result in wide‐reaching ecosystem changes. Predicting and addressing the additive effects of multiple, management actions are difficult. Once they occur, however, they require flexible conservation strategies that encourage the coexistence of people and predators (Chapron et al., 2014) and better link the comanagement of predators and prey.

## CONFLICT OF INTEREST

None declared.

## AUTHOR CONTRIBUTIONS

LME, HQ, and DC conceived the project and conducted the research. LM conducted the CMR analyses, with input from HW and LME. LME, LM, and HW drafted the manuscript, and HQ and DC contributed feedback. All authors approved the final version.

## DATA ACCESSIBILITY

Data will be archived in Dryad.
