# Supplementary material for: Multiple anthropogenic interventions drive puma survival following wolf recovery in the Greater Yellowstone Ecosystem
Source: Ecol Evol. 2018 Jun 25;8(14):7236–45. doi: 10.1002/ece3.4264 (PMC6065371; doi:10.1002/ece3.4264)
Supplement: Supplementary file 1 [file ECE3-8-7236-s001.docx]

**Appendices for: Elbroch et al. XX. Ecology and Society**

**
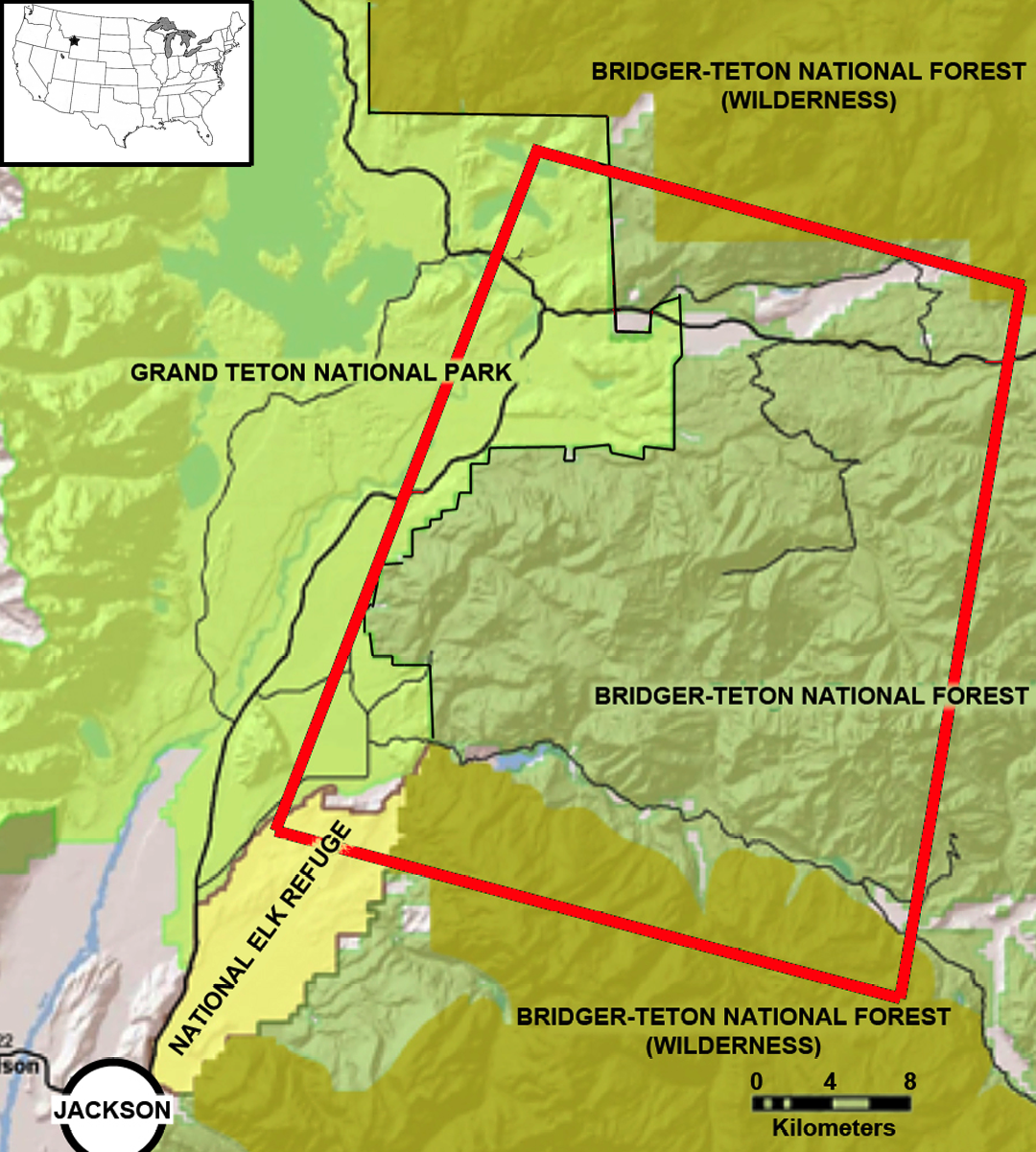
Appendix 1.** Figure A1.1: Location of the general study area in the United States and northwest Wyoming, USA (upper left). The finer scale map exhibits land ownership, and our primary puma capture area for and a close up of land ownership and our core puma study area in red.

**Appendix 2.** Table A2.1. Minimum number of wolves in our study area as compiled from US Fish & Wildlife Service Reports (2003-2015).

| **Wolf pack** | **2001** | **2002** | **2003** | **2004** | **2005** | **2006** | **2007** | **2008** | **2009** | **2010** | **2011** | **2012** | **2013** | **2014** | **2015** |
| --- | --- | --- | --- | --- | --- | --- | --- | --- | --- | --- | --- | --- | --- | --- | --- |
| Antelope |  |  |  |  |  | - | - | - | 8 | 5 | 4 | - | 2 |  |  |
| Black butte |  |  |  |  |  | - | 7 | 2 | 2 | 3 | 2 | 4 | 2 |  |  |
| Black rock |  |  |  |  |  |  |  |  |  |  |  |  | 7 | 4 | 4 |
| Buffalo |  |  |  |  |  | - | 13 | 7 | 9 | 22 | 14 | - | - |  |  |
| Flat Creek |  |  |  |  |  | 8 | 6 | - | - | - | - | - | - |  |  |
| Green River |  |  | 2 | 3 | 3 | - | - | 6 | 3 | 8 | 8 | 7 | 4 | 5 | 7 |
| Gros Ventre | 6 | 3 | 3 |  |  | - | 6 | 13 | - | 3 | - | - | - |  |  |
| Huckleberry |  |  |  |  |  | - | 7 | 5 | 3 | - | 9 | 6 | 7 | 11 | 3 |
| Lava Mountain |  |  |  |  |  | - | - | - | 3 | 7 | 10 | 3 | 10 | 15 | 24 |
| Lower Gros Ventre |  |  |  |  |  | - | - | - | - | - | - | 3 | 5 | 2 | 4 |
| Lower Slide |  |  |  |  |  |  |  |  |  |  |  |  |  | 2 | 2 |
| New Fork |  |  |  |  |  | - | - | - | - | - | 2 | 4 | - |  |  |
| Pacific Creek |  |  |  |  |  | 11 | - | 13 | 13 | 14 | 12 | 12 | 13 |  | 6 |
| Phantom Springs |  |  |  |  |  | - | - | - | 9 | 9 | 9 | 13 | 8 | 6 | 5 |
| Pinnacle Peak |  |  |  |  |  | - | - | 6 | 14 | 14 | 11 | 13 | 10 | 12 | 12 |
| Rim |  |  |  |  |  | - | - | - | 6 | 6 | 4 | 5 | 3 | 2 |  |
| Snake River |  |  |  |  |  | - | 9 | 11 | 4 | - | - | 7 | 4 |  |  |
| Teton | 4 | 12 | 14 | 12 | 13 | 11 | 3 | 8 | - | - | - | - | - |  |  |
| Togwotee |  |  |  |  |  | - | 7 | 10 | - | - | - | - | - |  |  |
| Upper Gros Ventre |  |  |  |  |  | - | - | - | - | - | - | 6 | 4 |  |  |
| Totals | 10 | 15 | 19 | 15 | 16 | 30 | 58 | 81 | 74 | 91 | 85 | 83 | 79 | 59 | 67 |

**Literature Cited**

U.S. Fish and Wildlife Service *et al.* (2004) *Rocky Mountain wolf recovery 2003 annual report*. Helena, Montana, U. S. Fish and Wildlife Service, USA.

U.S. Fish and Wildlife Service *et al.* (2005) *Rocky Mountain wolf recovery 2004 annual report*. Helena, Montana, U. S. Fish and Wildlife Service, USA.

U.S. Fish and Wildlife Service *et al.* (2006) *Rocky Mountain wolf recovery 2005 annual report*. Helena, Montana, U. S. Fish and Wildlife Service, USA.

U.S. Fish and Wildlife Service *et al.* (2007) *Rocky Mountain wolf recovery 2006 annual report*. Helena, Montana, U. S. Fish and Wildlife Service, USA.

U.S. Fish and Wildlife Service *et al.* (2008 *Rocky Mountain wolf recovery 2007 interagency annual report*. Helena, Montana, U. S. Fish and Wildlife Service, USA.

U.S. Fish and Wildlife Service *et al.* (2009) *Rocky Mountain wolf recovery 2008 interagency annual report*. Helena, Montana, U. S. Fish and Wildlife Service, USA.

U.S. Fish and Wildlife Service *et al*. (2010) *Rocky Mountain wolf recovery 2009 interagency annual report*. Helena, Montana, U. S. Fish and Wildlife Service, USA.

U.S. Fish and Wildlife Service *et al*. (2011) *Rocky Mountain wolf recovery 2010 interagency annual report*. Helena, Montana, U. S. Fish and Wildlife Service, USA.

U.S. Fish and Wildlife Service *et al*. (2012) *Northern Rocky Mountain wolf recovery 2011 interagency annual report*. Helena, Montana, U. S. Fish and Wildlife Service, USA.

U.S. Fish and Wildlife Service *et al*. (2013) *Northern Rocky Mountain wolf recovery 2012 interagency annual report*. (M. D. Jimenez, and S. A. Becker, editors). Helena, Montana, U. S. Fish and Wildlife Service, USA.

U.S. Fish and Wildlife Service *et al*. (2014) *Northern Rocky Mountain wolf recovery 2013 interagency annual report*. (M. D. Jimenez, and S. A. Becker, editors). Helena, Montana, U. S. Fish and Wildlife Service, USA.

U.S. Fish and Wildlife Service *et al*. (2015) *Northern Rocky Mountain wolf recovery program 2014 interagency annual report*. (M. D. Jimenez, and S. A. Becker, editors). Helena, Montana, U. S. Fish and Wildlife Service, USA.

**Appendix 3:** Goodness-of-fit testing for multistate capture-mark-recapture models.

Assessing how well a general model fits available data is an important step of any modelling exercise, including survival analyses based on capture-mark-recapture (CMR) data (Choquet et al., 2009). Importantly, tests of goodness-of-fit (GOF) should be conducted prior to model selection to prevent drawing incorrect biological conclusions from biased survival estimates. Multistate CMR models rely on three major assumptions (Choquet et al., 2009): 1) that the fate of one individual is independent from that of another; 2) that the recapture probability of a newly marked individual is the same as that of previously captured individuals; and 3) that marking individuals does not affect their behaviour or fate. Any violation in these assumptions may lead to underestimating the variance of parameters and result in the selection of potentially biased and over-parameterized models (Choquet et al., 2009; Fletcher et al., 2012).

Note that GOF testing in multistate CMR models is not meant to assess the predictive power of a single model in particular, but instead informs us of discrepancies between the general model and the data. This is essential so that we select an appropriate list of candidate models that explain variation in our selection parameter (survival) and prevent bias in the estimates of survival and recapture probabilities. Therefore, prior to final model selection we the following GOF tests on our fully parameterized survival models to ensure the above assumptions were met:

1. Test 3G.SR for transient effects:

Test 3G.SR is aimed at detecting a potential lack-of-fit due to the presence of transient individuals that emigrate temporarily from the study area (Choquet et al., 2009). It is the equivalent of test 3SR for single state models which tests the underlying null hypothesis that “newly marked and previously marked individuals have the same recapture probability at any given occasion”. If the null hypothesis is rejected recently captured individual tend to be encountered less frequently than previous ones due to age-dependent fate of transients (Choquet et al., 2009). The directional tests comparing encounter histories between newly and previously marked individuals were not significant (Table S1).

2. Test 3G.SM

Test 3G.SM is a composite test based on the conjunction of three different null hypotheses: 1) that the time and state of the first recapture probability is the same between newly and previously marked individuals in the same state; 2) that there is no difference in the detection between individuals observed at a certain occasion in a given state that have been previously encountered, possibly in another state; 3) and that there are no differences in the expected time of first recapture for individuals in a given state at a certain occasion that have been encountered earlier possibly in another state and will be later encountered in another specific state. Results from this composite test are often difficult to interpret and cannot be used as a tool to determine the presence of individual detection heterogeneity (Peron et al., 2010). The composite tests 3G.SM was not significant (Table S1).

3. Test 2M.LTEC

Test 2M.LTEC tests the null hypothesis that there is not a difference in the expected time and state of the next encounter of individuals with the same state on occasion i that were not encountered on occasion i+1 despite being alive at i+2 (Choquet et al., 2009). Test 2M.LTEC was not significant (Table S1).

4. Test M.ITEC for trap-response:

Test M.ITEC tests the null hypothesis that recapture probabilities of individuals in different states are independent of the previous encounter event (whether it was previously captured or not in the same state). If the test is significant, some individuals must respond positively or negatively to the capturing method (e.g., baited camera traps or individual afraid of flashing cameras). For CMR models based on telemetry data a “trap-happiness” response is likely to occur since collared individuals have higher probabilities of being recaptured. However, the relationship is expected to decrease with the life expectancy of the collar battery. We did find significant evidence of trap response effects (Table S1) due to combing location data from individuals fitted with VHF and GPS collars.

Assessing bias in survival due to trap-response effects:

Based on a simulation study, Nichols et al. (1984) reported that trap responses do not bias survival estimates but may decrease the precision of parameters. We followed methods presented by Pradel & Sans-Aguilar (2012) to reduce bias of age-specific survival estimates due to trap-responses and to compare the recapture probabilities of “trap aware” individuals previously marked from the recapture probabilities of “trap-unaware” individuals that were newly marked. We found a relative difference of 4.6E-05, -3.1E-06 and 8.9E-06 in survival probability of kittens, juveniles and adult respectively for the model ignoring the “trap awareness” status of individuals in the recapture probabilities. Such low relative biases indicated that the presence of the trap-response due to individuals fitted with collars did not affect our results.

Assessing individual detection heterogeneity:

Individual detection heterogeneity is quite common in CMR monitoring design and is known as one of the major cause of biased survival probabilities (Fletcher et al., 2012). Detection heterogeneity can be related to behavioural differences among individuals or study design (e.g., combining different method for marking individuals). Currently, there is no test available to account for detection heterogeneity in multistate models (Peron et al., 2012).

Assuming that such heterogeneity in our data (if any) would likely be related to our monitoring method, we also considered models accounting for heterogeneity in recapture probabilities among groups of individuals fitted with GPS collars, VHF collars or monitored via visual observations (very young kittens). Models accounting for individual detection heterogeneity related to the monitoring method were less supported by our data than the equivalent model with constant recapture probability (see appendix S3 model *φ*(season), F(i), p(marktype) and model *φ*(season), F(i), p(i)) and revealed identical survival estimates. To avoid issues associated with parameter identification, we did not include a covariate accounting for monitoring devices in our more parameterized models of cause-specific mortality rates.

Assessing the independent fate of kittens and other potential source of over-dispersion:

Finally we determined whether any observed lack-of fit may have resulted from over-dispersion in the data, which could be related to the possible dependent fates of kittens born in the same litter (Ruth et al., 2011). To do so we made sure that the variance inflation factor never exceeded a ratio of 3. We found slight evidence of over dispersion among age groups and more specifically among adults. Therefore, we corrected the deviance using the QAIC criterion that penalized models impacted by over dispersion (Choquet et al., 2009).

**References:**

Choquet, R., Lebreton, J. D., Gimenez, O., Reboulet, A. M. & Pradel, R. (2009) U CARE: Utilities for performing goodness of fit tests and manipulating Capture–Recapture data. *Ecography,* 32, 1071-1074.

Fletcher, D., Lebreton, J. D., Marescot, L., Schaub, M., Gimenez, O., Dawson, S. & Slooten, E. (2012) Bias in estimation of adult survival and asymptotic population growth rate caused by undetected capture heterogeneity. *Methods in Ecology and Evolution,* 3, 206-216.

Nichols, J. D., Hines, J. E. & Pollock, K. H. (1984) Effects of permanent trap response in capture probability on Jolly–Seber capture–recapture model estimates. *Journal of Wildlife Management,* 48, 289–294.

Péron, G., Crochet, P. A., Choquet, R., Pradel, R., Lebreton, J. D. & Gimenez, O. (2010) Capture–recapture models with heterogeneity to study survival senescence in the wild. *Oikos,* 119, 524-532.

Pradel, R. & Sanz-Aguilar, A. (2012) Modeling trap-awareness and related phenomena in capture-recapture studies. *PLoS ONE,* 7, e32666.

Ruth, T. K., Haroldson, M. A., Murphy, K. M., Buotte, P. C., Hornocker, M. G. & Quigley, H. B. (2011) Cougar survival and source–sink structure on Greater Yellowstone’s Northern Range. *Journal of Wildlife Management,* 75,1381-1398.

**Table A3.1**. Results of the Goodness-of-Fit for our Multistate capture-recapture data set combining live encounter and dead recoveries.

|  | Age group | Pearson statistics | pval | df |  |
| --- | --- | --- | --- | --- | --- |
| TEST 3G.SR | Kitten | 0.79 | 0.68 | 2 | Non-significant |
|  | Juvenile | 0.00 | 1.00 | 1 | Non-significant |
|  | Adult | 0.00 | 1.00 | 1 | Non-significant |
| TEST 3G.SM | Kitten | 21.01 | 0.10 | 14 | Non-significant |
|  | Juvenile | 2.58 | 0.77 | 5 | Non-significant |
|  | Adult | 2.38 | 0.88 | 6 | Non-significant |
| TEST M.LTEC | Kitten | 2.00 | 0.16 | 1 | Non-significant |
|  | Juvenile | 6.75 | 0.15 | 4 | Non-significant |
|  | Adult | 6.00 | 0.11 | 3 | Non-significant |
| TEST M.ITEC | Kitten | 22.64 | 0.02 | 11 | Significant |
|  | Juvenile | 35.28 | 0.04 | 22 | Significant |
|  | Adult | 84.32 | 0.00 | 24 | Significant |

**Appendix 4:** Table A4.1. Annual puma, wolf and elk estimates. We included both total elk and elk off the National Elk Refuge (NER) in our model comparisons.

| Year | Adult puma  Density per 890 km^2^ | Adult puma  Density per 100 km^2^ | Adult+dependents  density per 890 km^2^ | Adult+ dependents  density per 100 km^2^ | Annual wolf  estimates | Annual elk  estimates | Elk On NER | Elk Off refuge |
| --- | --- | --- | --- | --- | --- | --- | --- | --- |
| 2001 | - | - | - | - | 10 | 15,200 | 6128 | 9,072 |
| 2002 | 5.8 | 0.65 | 15.7 | 1.76 | 15 | 14,956 | 6366 | 8,590 |
| 2003 | 5.7 | 0.64 | 13 | 1.46 | 19 | 13,457 | 6992 | 6,465 |
| 2004 | 8.9 | 1.00 | 15.9 | 1.79 | 15 | 13,730 | 5876 | 7,854 |
| 2005 | 7.3 | 0.82 | 15.7 | 1.76 | 16 | 12,610 | 4969 | 7,641 |
| 2006 | 4.7 | 0.53 | 12 | 1.35 | 30 | 12,855 | 6730 | 6,125 |
| 2007 | 2.5 | 0.28 | 6.6 | 0.74 | 58 | 12,777 | 7279 | 5,498 |
| 2008 | 4.8 | 0.54 | 8.4 | 0.94 | 81 | 12,550 | 7947 | 4,603 |
| 2009 | 3.6 | 0.40 | 5 | 0.56 | 74 | 12,550 | 7269 | 5,281 |
| 2010 | 2.6 | 0.29 | 3.1 | 0.35 | 91 | 11,691 | 4348 | 7,343 |
| 2011 | 4.2 | 0.47 | 10.8 | 1.21 | 85 | 11,978 | 7746 | 4,232 |
| 2012 | 4.3 | 0.48 | 8.5 | 0.96 | 83 | 11,982 | 7360 | 4,622 |
| 2013 | 3.9 | 0.44 | 8.3 | 0.93 | 79 | 11,200 | 6285 | 4,915 |
| 2014 | 3.2 | 0.36 | 8.4 | 0.94 | 59 | 11,600 | 8296 | 3,304 |
| 2015 | 3 | 0.34 | 5.1 | 0.34 | 67 | 11,000 | 8390 | 2,610 |
